# Early detection of left ventricular diastolic dysfunction in Chagas' disease

**DOI:** 10.1186/1476-7120-4-18

**Published:** 2006-03-31

**Authors:** Tomás F Cianciulli, Jorge A Lax, María C Saccheri, Alonso Papantoniou, Luis A Morita, Nilda G Prado, Adriana N Dorelle, Adelina R Riarte, Horacio A Prezioso

**Affiliations:** 1 From the Division of Cardiology, Hospital del Gobierno de la Ciudad de Buenos Aires "Dr. Cosme Argerich", Buenos Aires, Argentina; 2 Instituto Nacional de Parasitología "Dr. Mario Fatala Chaben", Buenos Aires, Argentina; 3 Researchers of the Secretary of Health, Government of the City of Buenos Aires, Argentina

## Abstract

**Background:**

Chagas' disease may cause left ventricular diastolic dysfunction and its early detection in asymptomatic patients would allow to stratify the risk and to optimize medical treatment. The aim of this study is to investigate if transmitral Doppler flow can detect early abnormalities of the diastolic left ventricular function in patients during the indeterminate phase of Chagas' disease, in which the electrocardiogram (ECG), chest x-ray and 2-D echocardiogram (2D-echo) are normal.

**Methods:**

a group of 54 patients with Chagas' disease was studied and compared to a control group of 27 subjects of similar age. All were assessed with an ECG, chest X-ray, 2-D echo, and transmitral Doppler flow.

**Results:**

both groups had similar values in the 2D-echo. In patients with Chagas' disease, the transmitral Doppler showed a higher peak A velocity (control group: 0.44 m/sec, Chagas group: 0.55 m/sec, p = 0.001), a lower E/A ratio (control group: 1.45, Chagas group: 1.22, p < 0.05), and a lengthening of the deceleration time of early diastolic filling (control: 138.7 ± 26.8 msec, Chagas group: 167.9 ± 34.6 msec, p = 001), thus revealing an early disorder of the diastolic left ventricular function in patients with Chagas' disease.

**Conclusion:**

in patients with Chagas' disease who are in the indeterminate phase, transmitral Doppler flow allowed to identify early abnormalities of the left ventricular diastolic function, which provide useful clinical information for prognostic stratification and treatment.

## Background

Chagas' disease is a protozoan infestation caused by *Trypanosoma cruzi *and is one of the most relevant public health problems and a major cause of heart failure and death in Latin America. More than 100 million people are estimated to be infected and at risk of acquiring the disease and more than 18 million have already acquired it, and it causes 50.000 deaths per year, especially among the poorer countries [1]. As a consequence of migrations from Latin America, a globalization of the disease is under way, and there currently are thousands of infected Latin Americans residing in the US and Europe [2,3]. In Argentina, 20% of the 2 million infected people develop Chagas' disease, which causes 5.000 – 6.000 deaths per year.

Most infested patients are in the indeterminate phase of the disease, defined as the phase without ECG, X-ray or 2D-echo abnormalities and without signs of clinical cardiac involvement [4]; One third of these patients will eventually develop chronic Chagas cardiomyopathy [5]. During this indeterminate stage, the early detection of myocardial damage may be important for an individual approach to risk stratification. Among the available methods, transmitral Doppler flow may be very useful for the quantitative analysis of the left ventricular diastolic function.

The objective of this study was to assess the usefulness of transmitral Doppler flow to detect early left ventricular diastolic function abnormalities in patients during the indeterminate stage of Chagas' disease, when the ECG, chest X-ray and 2- echo are normal.

## Methods

### Population

We carried out a cross-sectional study of the population, including data collected from February 2004 until February 2006, from 81 subjects (46 men and 35 women) aged 41.4 ± 5.6 years (range, 30–50 years), whose ECG, chest X-ray and 2-D echo were normal. Subjects were divided into 2 groups: a Chagas group (54 patients with the indeterminate phase of Chagas' disease) and a control group (27 normal subjects). All patients were referred from the Instituto Nacional de Parasitología "Dr. Mario Fatala Chaben", in Buenos Aires. After patients were explained the goal of this investigation, all of them signed the informed consent.

The study was approved by our Institutional Review Board and the Investigation Health Council of the Secretary of Health of the Government of the City of Buenos Aires.

All patients with Chagas' disease, 25 men and 29 women, aged 41.7 ± 5.7 years (range 30–50 years), had a history of residence in an endemic zone and were asymptomatic.

Exclusion criteria were: coronary artery disease, valvular, myocardial or pericardial disease, congenital heart disease, clinical evidence of heart failure or asymptomatic systolic dysfunction (ejection fraction < 50%), hypertension, diabetes mellitus, anemia, asthma, chronic obstructive pulmonary disease, thyroid dysfunction, renal failure, pregnancy, a history of alcohol intake or other disorders that could potentially cause cardiac disease.

Left ventricular (LV) dilatation was a reason for exclusion of patients when the diastolic diameter of the LV exceeded 3.2 cm/m^2^, and similarly for systolic dysfunction when shortening fraction was below 28%.

Prior to selection, patients underwent an anamnesis, physical exam, ECG, chest X-ray, cardiac Doppler-echo and laboratory blood tests (complete blood count, sedimentation rate, blood urea nitrogen, creatinine, glycemia, ionogram, T3, T4, TSH and serology for Chagas' disease).

Immunodiagnostic tests were considered reactive for Chagas' disease in the following cases: enzymatic assay > 0.200, quantitative indirect hemagglutination > 1:32, quantitative indirect immunofluorescence > 1:32. Patients in whom 2 of these 3 tests were positive for Trypanosoma cruzi, were considered to have Chagas' disease.

### Control group

The control group consisted of 27 healthy volunteers, 21 men and 6 women, with a mean age of 40.5 ± 5.2 years (range 30–48 years), no history of endemic environment for South American trypanosomiasis, negative serology for Chagas' disease, normal physical exam, ECG, chest X-ray and Doppler-echo, and with similar exclusion criteria.

### Two-dimensional echocardiography

Doppler-echocardiograms were performed with Philips ultrasound equipment and a 2.5 MHz transducer. Parasternal (long- and short-axis views) and apical views were obtained (four-chamber, two-chamber and apical long-axis views).

All studies were recorded on videotape for their deferred analysis. Echocardiograms were reviewed by 2 experienced readers who were not aware of the serum test results for Chagas' disease.

The M-mode was derived from the short-axis view at the papillary muscle level, and the following parameters were measured according to the American Society of Echocardiography guidelines [6]: LV end diastolic diameter (LVEDd), LV end systolic diameter (LVESd), septal and posterior wall thickness, and antero-posterior diameter of the left atrium, measured from the left parasternal long-axis. The LV shortening fraction was calculated with the following formula: [(LVEDd-LVESd)/LVEDd] × 100.

The LV mass index was calculated according to Devereux's formula [7], and hypertrophy was defined as a value > 134 g/m^2 ^for men and > 110 g/m^2 ^for women.

### Transmitral flow velocity

**Figure 1:**
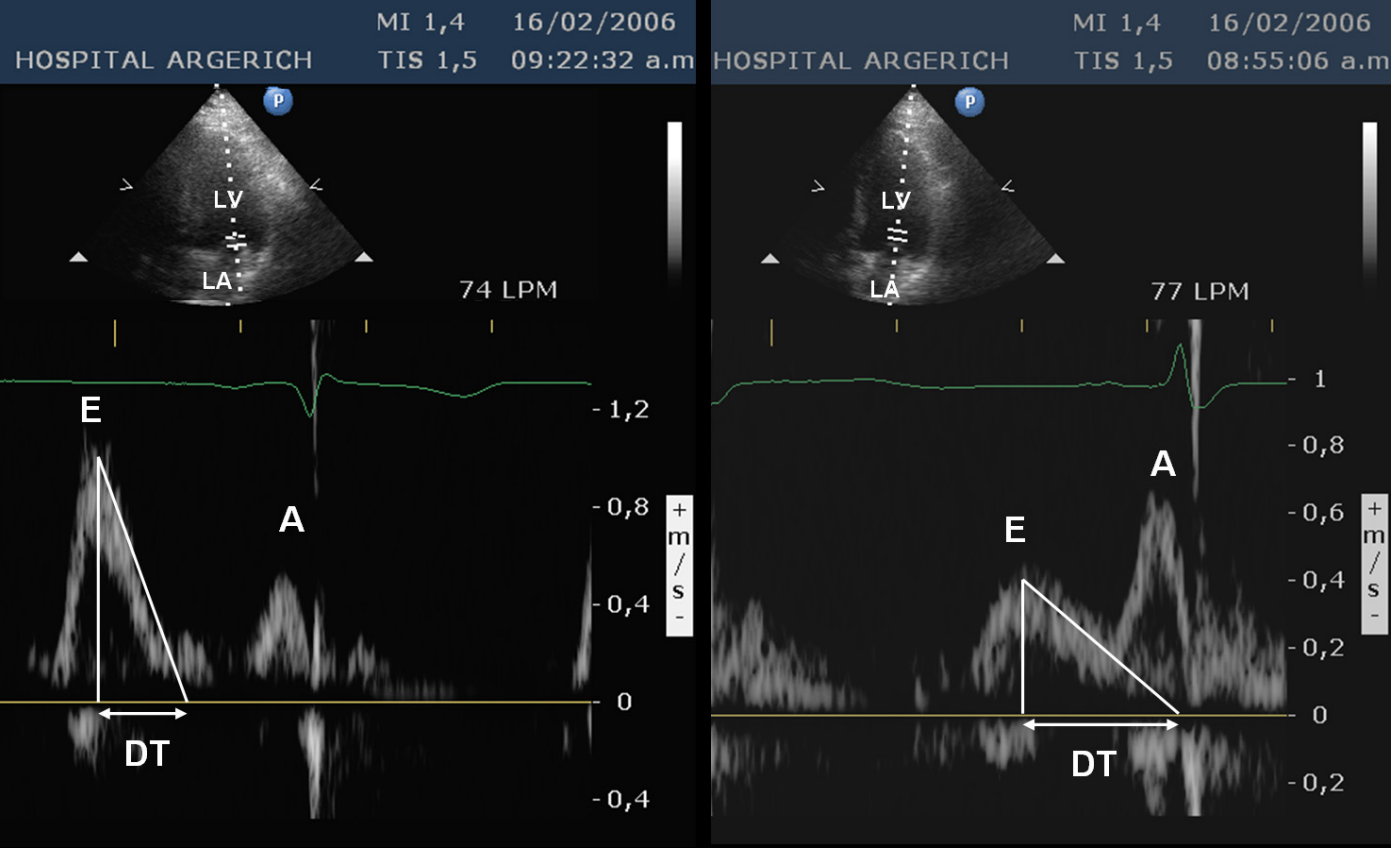
Apical four-chamber view of the left ventricle and Doppler transmitral flow velocity. Left: normal pattern. Right: abnormal pattern of a patient with Chagas' disease. LV: left ventricle, LA: left atrium, E: peak early diastolic velocity, A: peak late diastolic velocity, DT: deceleration time of the early diastolic flow.

Transmitral flow velocity was used to quantify left ventricular diastolic function (Figure 1). The sample volume was placed at the tip of the mitral valve leaflets in the four-chamber apical view. Peak early diastolic velocity (E-wave), peak late diastolic velocity (A-wave), E/A ratio and early filling deceleration time were measured [8].

All studies were recorded on videotape and later analyzed by two independent investigators who were unaware of the patients' clinical data. For each quantitative parameter, three consecutive beats were averaged.

### Statistical analysis

Quantitative data that were normally distributed were expressed as means ± standard deviation, and data with a non-gaussian distribution were expressed as medians (inter-quartile interval).

The comparison of inter-group quantitative variables was performed with a t-test in the case of variables with a normal distribution, and with the Wilcoxon Rank Sum Test in the case of variables with a non-normal distribution.

In 20 subjects, the intraclass correlation coefficient was used [9] to measure the intraobserver agreement between two measurements, and the interobserver agreement between the average of the intraobserver measurements and one interobserver measurement. A p value < 0.05 was considered to be statistically significant.

Statistical analyses were performed with the Statistix 7.0 software for Windows.

## Results

The clinical and echocardiographic characteristics of patients in the control and Chagas groups are summarized in Table 1. No statistically significant differences were found in age (control: 40.5 ± 5.2 years, Chagas group: 41.7 ± 5.7 years, p = NS), or body mass index (control: 26.04 ± 3.47 kg/m2, Chagas group: 27.30 ± 4.36 kg/m2, p = NS). Among Chagas patients, female sex prevailed (control 22.2%, Chagas group: 53.7%, p = 0.007).

**Table 1:** Clinical, echocardiographic and transmitral Doppler findings.

	**Control (n = 27)**	**Chagas (n = 54)**	**p**
**Age (y)**	40.5 ± 5.2	41.7 ± 5.7	NS
**Female, n (%)**	6 (22,2)	29 (53,7)	0.01
**Body mass index (Kg/m2)**	26.04 ± 3.47	27.30 ± 4.36	NS
**LVEDd (mm)**	49.3 ± 4.4	48.6 ± 4.5	NS
**LVESd (mm)**	28.6 ± 4.7	29.3 ± 4.3	NS
**LVEDd index (mm/m2)**	25.83 ± 3.10	26.64 ± 2.50	NS
**Fractional shortening (%)**	42.0 ± 6.9	39.8 ± 5.9	NS
**IVS (mm)**	9.6 ± 1.35	9.9 ± 1.6	NS
**PW (mm)**	7.59 ± 1.08	7.96 ± 0.84	NS
**LV mass index (g/m^2^)**	89.25 ± 16.97	95.78 ± 18.38	NS
**LA (mm)**	35.2 ± 3.5	34.8 ± 4.2	NS
**Aortic root (mm)**	31.3 ± 2.9	31.3 ± 3.2	NS
**E wave (m/sec)**	0.65 (0.58–0.73)	0.71 (0.58–0.79)	NS
**A wave (m/sec)**	0.44 (0.41–0.53)	0.55 (0.46–0.64)	0.001
**E/A ratio**	1.45 (1.19–1.57)	1.22 (0.99–1.52)	0.05
**DT (msec)**	138.7 ± 26.8	167.9 ± 34.6	0.001

LVEDd: left ventricular end diastolic dimension, LVESd: left ventricular end systolic dimension, IVS: interventricular septum, PW: posterior wall, LA: left atrial dimension, LV: left ventricle, A: peak early diastolic velocity, E: peak late diastolic velocity, DT: early filling deceleration time.

Both groups had normal dimensions, thickness and ventricular function, and no wall motion abnormalities were detected.

**Figure 2:**
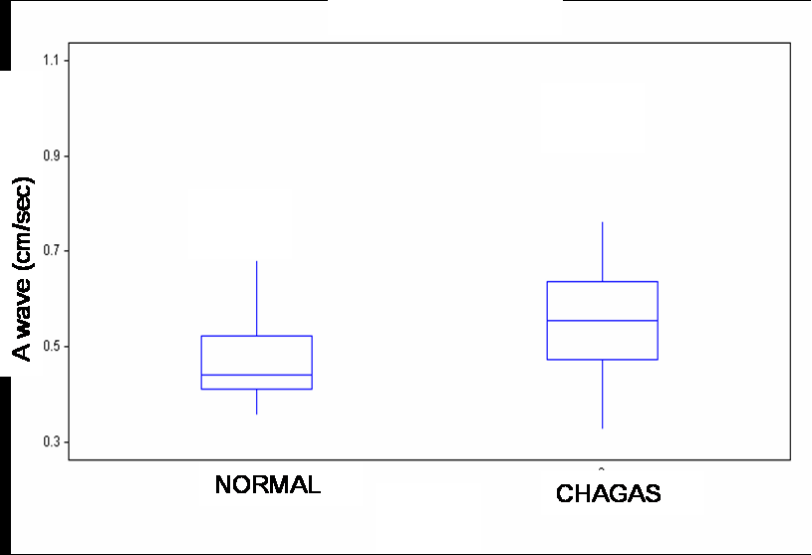
Late diastolic filling (A wave) values on transmitral Doppler in healthy subjects and patients with Chagas' disease. Box plot displaying median and interquartile range: solid circles indicate means.

**Figure 3:**
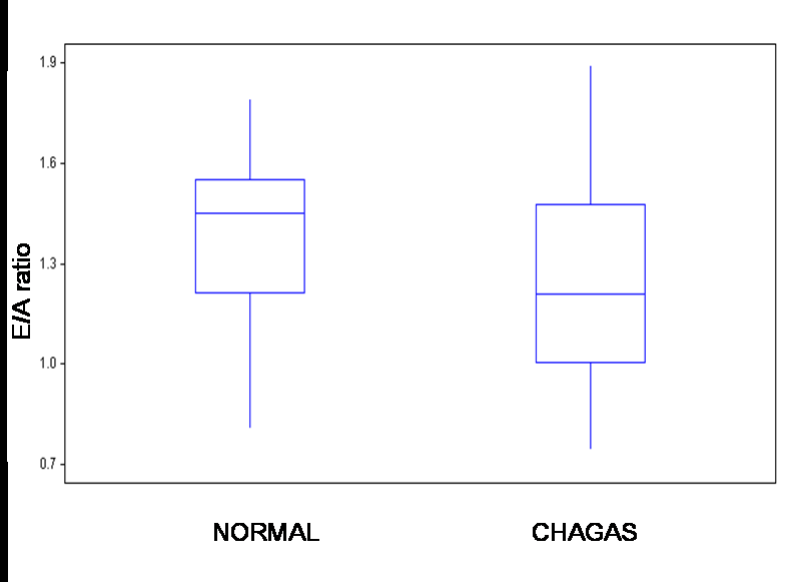
E/A ratio of the transmitral Doppler values in healthy subjects and patients with Chagas' disease. Box plot displaying median and interquartile range: solid circles indicate means.

**Figure 4:**
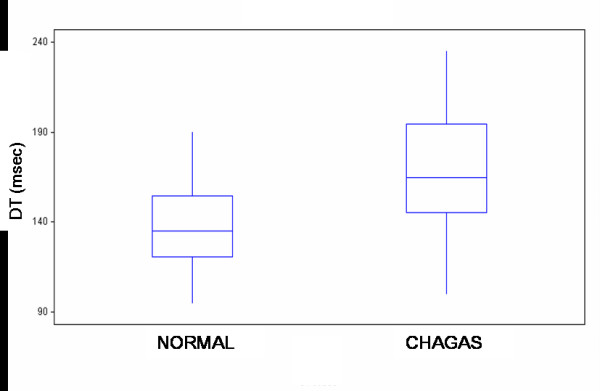
Deceleration time of the early diastolic filling values on transmitral Doppler in healthy subjects and patients with Chagas' disease. Box plot displaying median and interquartile range: solid circles indicate means.

By contrast, a statistically significant difference was seen between the two groups (Table 2 and Figures 2, 3, 4) in transmitral A peak velocity (control 0.44 cm/sec, Chagas group: 0.55 cm/sec, p = 0.001), in the mitral E/A ratio (control 1.45, Chagas group: 1.22, p < 0.05), and in the deceleration time of the early diastolic filling (control: 138.7 ± 26.8 msec, Chagas group: 167.9 ± 34.6 msec, p = 001), thus demonstrating an early disorder of the left ventricular diastolic function in patients with Chagas' disease.

### Intraobserver and interobserver agreement of transmitral Doppler flow measurements

Intraobserver agreement measured by the intraclass correlation coefficient varied between 0.995 for the velocities of both ventricles and 0.882 for the time measurements. Interobserver agreement varied between 0.993 for velocities and 0.832 for the time measurements.

## Discussion

Chagas' disease affects mainly the heart and may cause arrhythmias, systemic embolism, heart failure and sudden arrhythmic death. It consists of three phases: acute, indeterminate and chronic. The *acute *phase lasts 1 to 2 months, is generally mild and with few symptoms, and hence in most cases goes unnoticed; occasionally, especially in children, it may be fatal. The *indeterminate *phase, also called pre-clinical or latent phase, begins with the remission of the acute phase and ends with the first clinical manifestation of Chagas cardiomyopathy; it is generally benign but may develop serious, potentially lethal complications [10] and is characterized by a long period (20 to 30 years) during which the patient has positive serology but is asymptomatic, with normal ECG, chest X-ray and 2-D echo. One third of such patients progress to the *chronic *stage, developing chronic Chagas cardiomyopathy (ECG and echocardiographic alterations and heart failure).

When the parasite enters the human body, an inflammatory reaction takes place in the heart or the gastrointestinal system. As a consequence, an immune reaction ensues: when appropriate, it controls the parasitemia and the patient remains in the indeterminate phase. If, however, the immune reaction is inappropriate, parasitemia persists, inflammation progresses and with time (2 o 3 decades), there is damage to the heart, the digestive system or both.

Although most of the follow-up studies show that prognosis of patients in the indeterminate phase is good, every year 3% of them progress towards the chronic phase [5], which explains why, at 10 years, 30% of patients develop ECG or echocardiographic abnormalities. Additionally, sudden death may occur as the first manifestation of the disease in asymptomatic patients [11]. Therefore, an early identification of the signs of myocardial damage may be useful for risk stratification and the selection of an early medical treatment.

Barros et al [12] were the first authors to detect early LV diastolic dysfunction in patients with the indeterminate phase of Chagas' disease, demonstrated with transmitral Doppler flow, a lengthening of the deceleration time and isovolumic relaxation time, without changes in early and late ventricular filling velocities, but in our study we saw an increase in peak A velocity, a decrease in E/A ratio and an increase in the deceleration time of early diastolic filling, hence left ventricular diastolic dysfunction may be the earliest manifestation of heart disease and may occur in the absence of abnormalities in the overall LV systolic function; this might indicate a less affected population, which initially demonstrates only diastolic abnormalities.

Pathology data (biopsies and necropsies) have shown [13,14] early signs of myocardial damage with histological and ultrastructural changes in 30% to 60% of patients with Chagas disease and no evidence of heart disease, such as hypertrophy, degeneration of myocardial fibers, interstitial edema, fibrosis and an inflammatory infiltrate. These alterations are not serious enough to cause a disturbance in the conduction system or the contractile function which could be seen by ECG or 2D-echo, but they could be observed by using methods such as DTI that enable the evaluation of regional cardiac function.

This preclinical myocardial damage observed during the indeterminate phase of the disease is not an inactive stage but is progressive, and the progression continues from the destruction of myocardial cells until their replacement by fibrosis [4], which may be shown by cardiovascular magnetic resonance in up to 20% of patients who are in the indeterminate phase of Chagas' disease [15].

Myocarditis in Chagas' disease initially causes low grade focal damage which may be detected early with transmitral Doppler flow in patients with the indeterminate phase of Chagas' disease, while a more severe damage causes systolic myocardial dysfunction, evidenced by morphological and functional changes in the 2D-echo, characteristic of the chronic phase of the disease.

## Conclusion

In patients who are in the indeterminate phase of Chagas' disease, transmitral Doppler reveals early changes in left ventricular diastolic function. This evidences of preclinical myocardial damage and would hence allow to stratify the risk, to optimize medical treatment and improve the quality and duration of patients' lives. Prospective longitudinal cohort studies might allow to confirm the efficacy of transmitral Doppler in the evaluation of the natural history of patients with subclinical myocardial disorders.

## Competing interests

The authors declare that they have no competing interests. The manuscript has not been published and is not being considered for publication elsewhere in whole or in part in any language.

## Authors' contributions

TFC, JAL and LAM performed the echocardiographic images and participated in the manuscript description. ARR, NGP and attended the patient and prepared the manuscript. MCS, HAP and AP participated in the design and review of the manuscript. All authors read and approved the final manuscript.
